# The Program SI! intervention for enhancing a healthy lifestyle in preschoolers: first results from a cluster randomized trial

**DOI:** 10.1186/1471-2458-13-1208

**Published:** 2013-12-20

**Authors:** José L Peñalvo, Mercedes Sotos-Prieto, Gloria Santos-Beneit, Stuart Pocock, Juliana Redondo, Valentín Fuster

**Affiliations:** 1 Area of Epidemiology and Populations Genetics, Centro Nacional de Investigaciones Cardiovasculares (CNIC), Calle Melchor Fdez Almagro 3, Madrid 28029, Spain; 2 International SHE Foundation, Barcelona 08037, Spain; 3 Department of Medical Statistics, London School of Hygiene and Tropical Medicine, London WC1E 7HT, UK; 4 Department of Cardiology, Mount Sinai School of Medicine, New York, NY, USA

**Keywords:** Health education, Health promotion, Children’s health

## Abstract

**Background:**

Unhealthy lifestyles contribute to the development of cardiovascular risk factors, whose incidence is increasing among children and adolescents. The Program SI! is a long-term, multi-target behavioral intervention to promote healthy lifestyle habits in children through the school environment. The objective of the study is to evaluate the efficacy of this intervention in its first phase, preschoolers.

**Methods:**

Cluster-randomized controlled trial in public schools in the city of Madrid, Spain. A total 24 schools, including 2062 children (3–5 years), 1949 families, and 125 teachers participated in the study. Schools were assigned to their usual school curriculum or to engage in an additional multi-component intervention (Program SI!). The primary outcome of this trial is 1-school year changes from baseline in scores for children’s knowledge, attitudes and habits (KAH). Secondary outcomes are 1-school year changes from baseline in scores for knowledge, attitudes, and habits among parents, teachers, and the school environment.

**Results:**

After 1-school year, our results indicate that the Program SI! intervention increases children’s KAH scores, both overall (3.45, 95% CI, 1.84-5.05) and component-specific (Diet: 0.93, 95% CI, 0.12-1.75; Physical activity: 1.93, 95% CI, 1.17-2.69; Human body: 0.65, 95% CI, 0.07-1.24) score.

**Conclusions:**

The Program SI! is demonstrated as an effective and feasible strategy for increasing knowledge and improving lifestyle attitudes and habits among very young children.

**Trial registration:**

NCT01579708, Evaluation of the Program SI! for Preschool Education: A School-Based Randomized Controlled Trial (Preschool-SI!).

## Background

The increasing prevalence of cardiovascular disease (CVD) and its associated conditions is well documented for adults, but these rates are also rising among children and young adults [1]. The development of CVD risk factors carries a behavioral component that may be corrected at an early age, when behaviors are first formed, by effective health promotion initiatives [2]. Lifelong acquired behaviors are resistant to change, and therefore acquisition of healthy behaviors should begin as early in life as possible. Many reports analyzing cardiovascular health promotion strategies recommend school-based interventions as the most effective way to promote healthy behaviors from early childhood [3,4]. A successful school-based initiative should include not only a high-quality intervention but also a long-term sustainable system to gradually introduce children to healthy choices that can be learned and retained, resulting in a healthier life during adulthood. This sustainable intervention must also reach out to the children’s most proximal environment: their families, teachers and school.

Within this framework, the first phase of the Program SI! (Salud Integral = Comprehensive Health) targets children from 3 to 5 years of age, aiming to establish appropriate lifestyle behaviors early in life. Designed as a thorough, multilevel, school-based intervention, the project is pursuing a global vision of promoting cardiovascular health and preventing obesity. In this cluster-randomized controlled intervention trial, we aimed to evaluate the efficacy of the Program SI! for improving indicators of the acquisition of healthy behaviors in children aged 3–5.

## Methods

### Study design, setting, and participants

**Figure 1:**
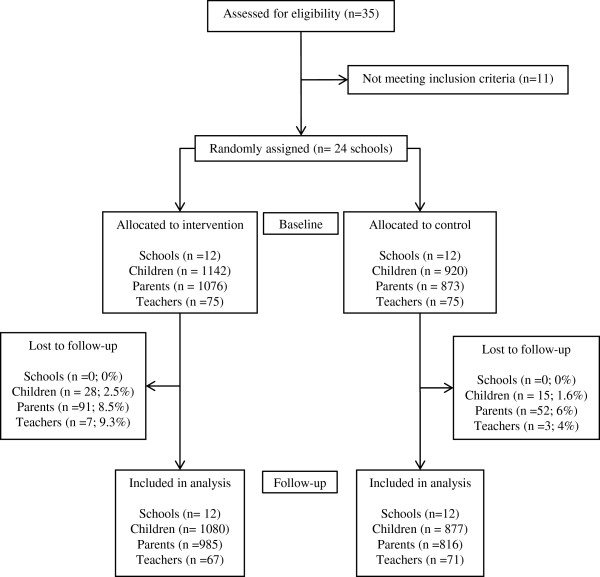
Flow chart for enrollment, baseline measurements and follow-up in the Program SI!.

We followed the updated CONSORT Statement for reporting cluster-randomized trials. The study was designed as a cluster-randomized, open label, parallel group, controlled trial. The detailed protocol of the study has been described previously [5]. Schools were randomized 1:1 to either intervention with Program SI! or to follow their usual curriculum. The study was based on a hierarchical design, where the schools were the units of randomization, intervention and analysis. The second level of analysis consisted of the school’s 3–5 year old children, their teachers, and parents. In previous school-based interventions, intra-class correlation (school-group effect) coefficients ranged from 0.05-0.30 for scores that measured academic results [6-8]. Taking this range into consideration, and using an estimated intra-class correlation of 0.15, we calculated 20 schools (50 children per class year) would be sufficient to detect effects with a power greater than 80%, and a chance of a type-I error of 0.05. In anticipation of possible losses during the study, a total of 24 schools were randomized, 12 in each group. Eligible schools were selected by applying a series of inclusion/exclusion criteria to obtain a final sample with comparable socio-economic characteristics. During the academic year 2010/11, the number of public schools in the area of Madrid was 787. Of these, approximately 20% of the centers (174 schools) were selected on the basis of the following inclusion criteria: be located in the city of Madrid, have canteen services available, and have two or more classrooms per class year (50 children per class year were needed). We distributed the schools according to percentages of students from an immigrant background and percentage of student receiving scholarships, and chose a subsample of 73 representing the mean values of the distribution [5]. These schools were invited to participate during a 1-day meeting at which the basic characteristics of the Program SI! intervention were explained, and 35 schools finally agreed to participate. The final 24 schools were chosen by excluding those with more than 2 classes per class year (Figure 1) avoiding large schools.

### Randomization

Schools meeting the inclusion criteria were randomized (1:1) according to quartiles of the percentage of children from immigrant families attending the school, in order to ensure that the two groups were balanced as regards of cultural profile. A blind randomization was carried out by running a randomization script over the list of encrypted codes of the schools. Allocation sequence, school’s enrollment and assignment to intervention were performed by the study investigators. Informed written consent to participation was required from the parents or legal guardians on behalf of their children, as well as from the teachers. The information collected was treated according to Spanish Law 15/1999 for the Protection of Personal Data. A data encryption system was used to guarantee the confidentiality of the information provided. The Madrid Regional Committee for Clinical Research (CEIC-R) approved the study.

### Intervention

The Program SI! was conceived as a permanently evolving intervention, with continuing improvements in materials and strategies introduced on the basis of the evaluation’s results [5,9]. All materials, contents and activities were developed by experts from different knowledge areas (education, psychology, medicine, biology, physical activity and nutrition), and pilot-tested before implementation [5]. The intervention seeks to promote cardiovascular health through the acquisition of healthy behaviors relating to four lifestyle components: dietary habits, physical activity patterns, human body and heart, and management of emotions. This comprehensive intervention was adapted from our previous experience in Colombia using the concept of social cognitive theory and trans-theoretical models in health promotion [9].

To obtain a quantitative measure of behavior modification, we used a composite score representing the five levels of the trans-theoretical model: aggregating the "pre – contemplative" and "contemplative" stages as the acquisition of knowledge (K), the "preparation" phase as to set this knowledge into attitudes (A), and the last stages "action" and "maintenance" as the acquisition of the desired habit (H). This was translated into component-specific KAH scores, plus an overall score representing the intervention as a whole. Finally, we recognized the relevance of the social cognitive theory by including intervention strategies towards parents and teachers, as well as the school environment to help achieving optimal results on behavior modification.

School’s adherence to the intervention is enhanced by the continuous support in each school from external (Program SI! coordinator) and in-house (school coordinator) supervisors that monitor all intervention processes and compliance with the minimum requirements. These two people broadcast all the information related to Program SI! to other teachers and to the parents, and ensure that the intervention is carried out throughout the school year [5]. These two coordinators work closely together and use a CRM (customer relationship management) model to help tracking all intervention-related activities. This allows Program SI! staff to collect metrics and report on the different processes.

For children, the Program SI! intervention uses classrooms materials (including different resources such as healthy tales, educational games and audio-visuals), take-home activities with their families, and activities organized within the school’s annual Health Fair. The classrooms materials include three teaching units for diet, physical activity and human body components (20 hours per component) and Sesame Street Emotion Cards to address managing emotions (10 hours per academic year). These cards work on self-awareness, self-esteem, managing emotions, autonomy, decision-making, acceptance and respect for others and their differences, and listening skills and communication, setting on the basis of protective factors against substance abuse in the future (smoking, alcohol and drugs) and potential psychological disorders (anxiety or depression) related with CVD.

To reach out to parents, the children take weekly a "Healthy tip" proposing a healthy activity for the family to share over the weekend (3 notes per component for a total of 12 notes). These activities are simple, and always focus on facilitating time and space for parents to interact with their children. Some examples include enjoy chatting while eating instead of watching TV, or go to the market and cook together with the children. With these suggestions, intervention aims to go beyond the simple transfer of information, although there is of course a large number of extra resources available for parents who are interested in learning more. Both parents and teachers have access to the password-restricted Program SI! resources through http://si.fundacionshe.org/portal/2313/programa_si.aspx, which contains additional information on the intervention.

At least one teacher on each school receives a certified training in the Program SI! contents and strategies (an expert-led 30-hour course). Teachers have access to the intervention website where they can download all the resources (guides of teaching units, tales, Sesame Street audiovisuals, workshops, games, etc.). They also have access to a blog where they can post pictures and comments about the intervention activities carried out throughout the course. They can also view and comment on the blogs of the other participating schools. On the website there is also a forum where they can discuss any aspect of the intervention and where the Program SI! coordinators are actively involved. These two tools are highly valued by teachers because they can share experiences and get ideas for activities and workshops. Likewise, training sessions facilitate the exchange of experiences and is very enriching not only for teachers but also for the coordinators in order to optimize the implementation and adherence to the program.

For the school environment, the intervention focuses on recommendations given to the principal and teachers (also available on the website). These recommendations encourage healthy practices such as promoting the replacement of candy on birthdays, provide spaces to solve conflicts between students, involve parents in school activities or improve the adequacy of playground for promotion of physical activity during recess.

### Control group

Schools in the control group were informed that the intervention entailed a health promotion initiative for children but were not aware of the specifics or the main objective of the intervention. Control schools follow their regular curriculum.

### Data collection

Children, parents, teachers and principals participating in the study were evaluated before the intervention and at the end of the school year. For children, parents and teachers we used an adapted version of the questionnaires, and their scoring system first developed in the Colombian Initiative for Healthy Heart Study [5,9]. Briefly, these questionnaires assess the knowledge (K), attitudes (A) and habits (H) of the study participants’ representing each of the components of the intervention (KAH-diet, KAH-physical activity, and KAH-human body). An overall score representing the intervention as a whole (overall KAH) was derived from the component-specific KAH for each population subgroup: children (39 items), parents (42 items), and teachers (30 items), all ranging from 0–80 [5,9]. For the evaluation of the fourth component of the intervention (management of emotions) in children, we used the Test of Emotional Comprehension (TEC) [10]. Children were evaluated by a team of trained psychologists [5].

To assess the school environment, schools’ principals filled out a questionnaire consisting on 10 items (score range 0–10) corresponding to the involvement of the center on intervention-related activities such as food items allowed for birthday celebrations, and specific canteen regulations. There are also questions concerning the availability and use of sports facilities and playgrounds, or the extra-curricular sport activities offered. Questions regarding the emotional management component concerned the use of video game consoles or electronic devices (which may isolate children) or the availability of specific spaces for conflict solving.

### Objectives and outcome

The primary aim was to evaluate the efficacy of Program SI! to change the knowledge, attitudes, and habits (KAH) of 3-5-year-old children, their parents, and teachers, in relation to lifestyle (diet, physical activity, human body, and emotion management) after 1 year of a school-based intervention.

### Statistical methods

To evaluate changes after intervention we conducted analyses in those children who have data for the primary outcome (overall score) at baseline and after 1-school year. Mixed linear models that take account of the cluster-randomized design were used to test for intervention effects. Fixed effects in each model were the corresponding baseline score, the class year, and the treatment group. Schools were handled as random effects. Subsequent models were fitted to identify possible age-by-treatment or sex-by-treatment interaction effects for the main outcome variable. Similar mixed models were constructed for the subcomponents of the primary endpoint in children (i.e. diet, physical activity, body and heart) and also for the secondary outcome variables in parents, teachers and the school environment. All analyses were performed on an intention-to-treat basis using STATA version 12 (STATACORP, College Station, Texas, USA).

## Results

The study included 12 intervention schools and 12 control schools, with 1142 and 920 children respectively during October 2011. In addition, a total of 1949 parents and 150 teachers were involved. After the intervention, during 1-school year (until June 2012), 2% of the children, 8% of parents, and 8% of teachers were lost to follow up or had incomplete data. No school withdrew from the trial during the study period (Figure 1). No adverse events or harms were reported. Table 1 contains the baseline information for children, parents, teachers, and schools for the intervention and control groups. Overall and component-specific baseline KAH scores are also presented. As expected by design, no differences were found between the two treatment groups at baseline.

**Table 1:** Baseline demographic characteristics of children, parents and teachers after school randomization

	**Control**	**Intervention**
**Schools**	12	12
**Children**	920 (44.6)	1142 (55.4)
Age, mean (SD)	3.8 (0.9)	3.7 (0.9)
Class year, N (%)		
Age 3	371 (40.3)	455 (39.9)
Age 4	286 (31.1)	354 (30.9)
Age 5	263 (28.6)	333 (29.2)
Gender, N girls (%)	440 (47.8)	581 (50.9)
KAH-D score, mean (SD)	19.2 (4.6)	18.7 (4.9)
KAH-PA score, mean (SD)	17.9 (4.7)	17.5 (4.7)
KA-BH score, mean (SD)	13.8 (5.2)	13.1 (5.3)
KAH overall score, mean (SD)	50.2 (10.5)	49.2 (10.8)
Emotions score, mean (SD)	3.3 (2.0)	2.9 (2.2)
**Parents**	872 (44.76)	1076 (55.2)
Age, mean (SD)	36.97 (5.78)	37.0 (5.8)
Gender, N females (%)	662 (75.9)	857 (79.6)
Salary, N (%)		
<22,500 €	490 (61.6)	640 (65.0)
>22,500 €	344 (35.0)	344 (35.0)
Education level, N (%)		
None/Elementary	56 (6.6)	100 (9.6)
Secondary school	63 (7.4)	85 (8.2)
High school	165 (19.4)	224 (21.6)
Technical training	168 (19.7)	196 (18.9)
University degree	399 (46.9)	434 (41.8)
Birthplace, N (%)		
Madrid	486 (56.9)	578 (54.6)
Rest of Spain	183 (21.4)	214 (20.3)
South America	127 (14.9)	196 (18.5)
Other countries	58 (6.8)	69 (6.6)
KAH-D score, mean (SD)	20.8 (4.0)	21.2 (3.7)
KAH-PA score, mean (SD)	20.1 (4.6)	20.5 (4.6)
KAH-HB score, mean (SD)	17.2 (3.0)	17.5 (2.8)
KAH overall score, mean (SD)	58.0 (9.3)	59.1 (8.8)
**Teachers**	75 (50.0)	75 (50.0)
Age, mean (SD)	43.5 (9.0)	42.7 (9.1)
Gender, N females (%)	74 (98.7)	71 (94.7)
KAH-D score, mean (SD)	22.7 (3.4)	22.1 (3.0)
KAH-PA score, mean (SD)	22.5 (3.6)	22.8 (3.9)
KAH-HB score, mean (SD)	17.3 (2.7)	17.8 (2.4)
KAH overall score, mean (SD)	62.5 (7.4)	62.7 (7.3)
**School environment score**	8.1 (1.6)	8.1 (2.5)

KAH-D: Diet knowledge, attitudes, and habits. KAH-PA: Physical activity knowledge, attitudes, and habits. KAH-BH: Body and heart knowledge, attitudes, and habits. Continuous variables are expressed as mean (standard deviation). Categorical variables are presented as N = sample size (percentage).

Table 2 presents the changes in overall and component-specific KAH scores for children by treatment group. Using a random effect model and after adjustment for class level and baseline score, children in the intervention group showed a significantly larger increase in all scores. The increase in the overall KAH score in the intervention group was 3.45 points higher than in the control group (95% CI: 1.84, 5.05; p < 0.001). All component-specific KAH scores also increased significantly more in intervened children, with the largest difference, 1.93 points, in the KAH-physical activity score (95% CI: 1.17, 2.69; p < 0.001). For the emotion component a small and non-significant increase was found (Table 2).

**Table 2:** Changes in individual and overall scores after 1 school year follow-up in children, parents and teachers

**Within group difference**					
**Children**	**Score range**	**Control (n = 877)**	**Intervention (n = 1080)**	Difference (95% CI)*	**p-value**
KAH-D	0-30	6.87 (6.41, 7.32)	8.19 (7.79, 8.60)	0.93 (0.12, 1.75)	0.025
KAH-PA	0-30	0.48 (0.10, 0.87)	2.81 (2.46, 3.16)	1.93 (1.17, 2.69)	<0.001
KA-BH	0-20	0.44 (0.04, 0.84)	1.28 (0.92, 1.64)	0.65 (0.07, 1.24)	0.029
KAH overall	0-80	10.49 (9.63, 11.35)	14.61 (13.87, 15.35)	3.45 (1.84, 5.05)	<0.001
Emotions	0-9	2.41 (2.30, 2.51)	2.57 (2.48, 2.66)	0.08 (-0.12, 0.29)	0.437
**Parents**	**Score range**	**Control (n = 816)**	**Intervention (n = 985)**	Difference (95% CI)*	**p-value**
KAH-D	0-30	-0.74 (-0.99, -0.5)	-0.49 (-0.72, -0.27)	0.08 (-0.28, 0.43)	0.664
KAH-PA	0-30	0.64 (0.31, 0.96)	0.88 (0.58, 1.17)	0.05 (-0.35, 0.45)	0.792
KA-health	0-20	0.04 (-0.15, 0.23)	0.16 (-0.02, 0.34)	0.03 (-0.20, 0.27)	0.783
KAH overall	0-80	-0.09 (-0.62, 0.44)	0.55 (0.08, 1.03)	0.30 (-0.33, 0.94)	0.351
**Teachers**	**Score range**	**Control (n = 71)**	**Intervention (n = 67)**	Difference (95% CI)*	**p-value**
KAH-D	0-30	-0.86 (-1.64, -0.08)	0.82 (0.06, 1.58)	1.23 (0.46, 2.03)	0.002
KAH-PA	0-30	0.83 (-0.18, 1.85)	0.79 (-0.33, 1.91)	0.18 (-0.82, 1.18)	0.724
KA-health	0-20	1.15 (0.46, 1.84)	1.0 (0.38, 1.61)	0.17 (-0.48, 0.81)	0.614
KAH overall	0-80	1.12 (-0.65, 2.90)	2.61 (0.66, 4.56)	1.48 (-0.30, 3.27)	0.103
**School environment**	**Score range**	**Control (n = 12)**	**Intervention (n = 12)**	Difference (95% CI)*	**p-value**
Overall score	0-10	1.02 (0.92, 1.13)	1.07 (0.97, 1.16)	0.005 (-0.07, 0.08)	0.882

KAH-D: Diet knowledge, attitudes, and habits. KAH-PA: Physical activity knowledge, attitudes, and habits. KAH-BH: Body and heart knowledge, attitudes, and habits. * Results from a mixed linear effect model, as explained in Statistical methods. Models adjust for baseline score and class year, and school is handled as a random effect.

**Figure 2:**
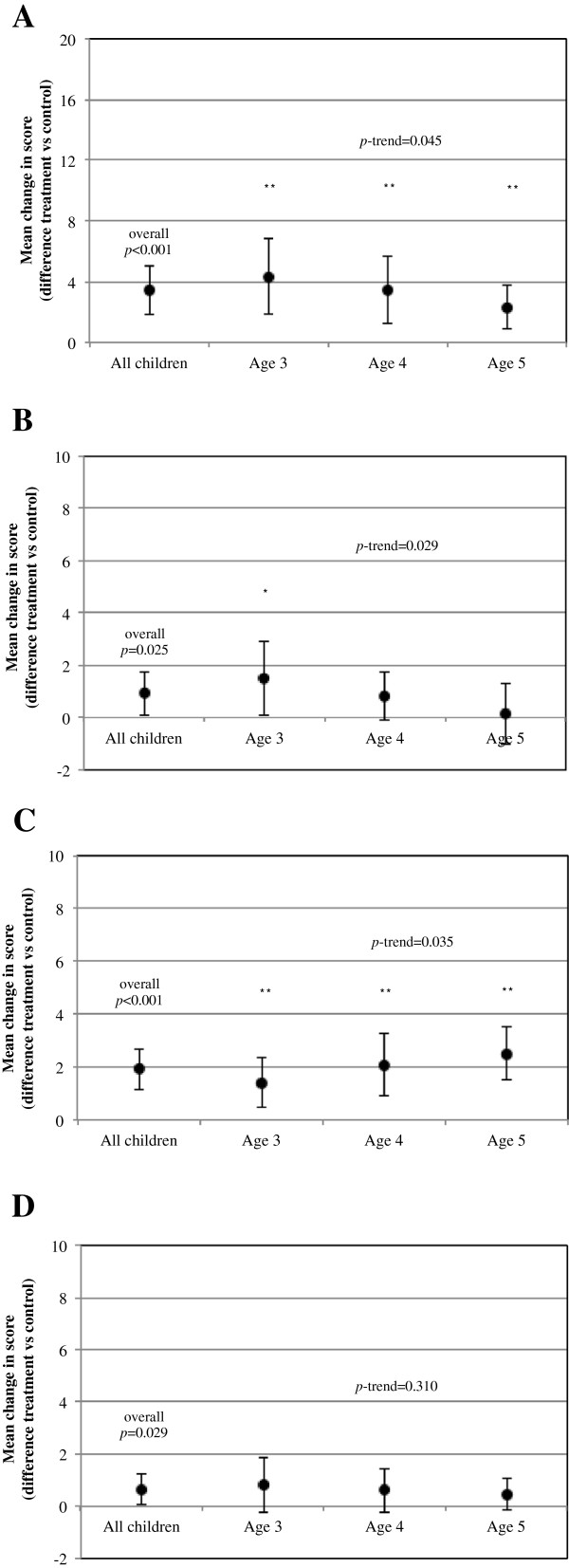
**Mean differences in knowledge, attitudes and habits (KAH) scores after 1-year follow-up in children (overall and by age). (A)** KAH-overall. **(B)** KAH-diet. **(C)** KAH-physical activity. **(D)** KAH-heart and body. P values relate to overall mean differences in score changes between intervention and control groups. P trend values test whether the treatment effect varies with children’s age (by class year). **P<0.005. *P<0.05.

Figure 2 displays the overall and component-specific KAH score differences (intervention versus control), stratified by children’s age. KAH-diet score was higher among the 3-year olds, and the impact seemed to be lower in older children (p-trend = 0.029); in contrast, the KAH-physical activity score in the intervention group appeared to increase with the age of the children (p-trend = 0.035). Children in the intervention group scored 0.65 (95% CI: 0.07, 1.24; p = 0,029) points more than controls in the KAH-body and heart component, although no interaction was detected with children’s age.

**Figure 3:**
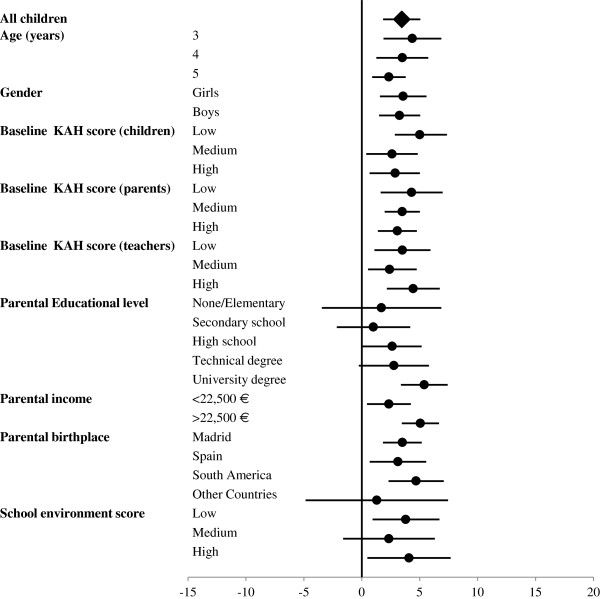
Mean differences (95% CI) in overall KAH score changes after 1-year follow-up between children in the intervention and control groups, according to selected variables.

A break down of the mean differences (intervention versus control) in overall KAH scores according to several variables (Figure 3) revealed a marked interaction with children’s age (p = 0.046), the largest differences being found among the youngest. Children starting from a lower baseline score also seem to benefit more from the intervention (p-for interaction = 0.051), and larger benefits were seen in children whose parents have a university degree (p-for interaction = 0.008), and higher economic status (p-for interaction = 0.011).

No effect was found on the overall scores for parents, teachers or the school environment (Table 2). The only component-specific score showing evidence of an effect in these groups was KAH-diet, which improved more in the intervened teachers.

## Discussion

Although many school-based intervention studies have been implemented, few randomized controlled trials have targeted very young children (3–5 years) [11-21]. In addition, other studies including older children focused mainly on one component of lifestyle (e.g. diet or physical activity) [8,11,15,19-31], and very few adopted a multicomponent approach to children’s health promotion, including the influence of the children’s environment [8,15,25,32].

After one year, children engaged in the multilevel, behavioral intervention, showed greater improvements (5.1% differential increase) than their control counterparts in their knowledge, attitude and habits in relation to three lifestyle-related components (diet, physical activity, and human body). These results are very much in line with our previous results in Colombia where, after a 6-month controlled evaluation trial involving 1216 children (3–5 years old) a 5.6% differential increase in the overall KAH score favorable to the intervened children was found. In this study, the largest effect was found for 4-year-old children [9]. In our study, the youngest children (age 3) seem to benefit most from the intervention, possibly reflecting different cultural influences in the two countries. The influence of the environment on children’s behavior is apparent in our study, where better results were seen on children from higher socio-economic level as well as other parental socio-demographic characteristics. This reinforces the need of impacting the children’s proximal environment to achieve maximum results [33,34]. In line with this, a recent review concluded that the most effective school-based interventions are those that include the family, and that center on realistic intermediate goals, such as changes in knowledge and attitudes towards the learning component (diet and physical activity, for instance), as early indicators of eventual improvements in adult cardiovascular health [2]. Like the Colombian Initiative [9], the Program SI! targets an early change in behavior an focuses intermediate indicators of health (KAH) rather than hard endpoints such as obesity. There are very few studies assessing these type of indicators in young children [11,33,35,36] but in all of them the results have been positive towards improved lifestyle behaviors with small effect on secondary anthropometric measures.

Accordingly, large studies assessing the impact of behavioral intervention on hard outcomes (e.g. obesity) among young children such as the Pathways Study [8], or the Healthy Study, have had limited success [37]. The authors concluded that to prevent the increasing levels of obesity in children, more intense and longer interventions would be necessary. In Europe, multi-country efforts like the IDEFICS study are trying to underpin this difficult process of achieving behavior change to reduce the prevalence of diet- and lifestyle-related diseases and disorders among children 2 to 9 years [18]. The results of this landmark study are currently being incorporated into various guidelines on nutritional, behavioral and lifestyle aspects. The Ballabeina Study, also involving preschoolers [16] has obtained significant benefits in the intervention group for physical activity, media use and eating habits [32]. Other European studies have demonstrated that even short dietary interventions in preschoolers may obtain a significant increase in children’s fruit and vegetable intake [14].

In this study, an additional but essential component to behavior change has been introduced for the first time as part of a comprehensive intervention. The correct management of emotions intends to provide very young children with tools to develop protective behaviors against substance abuse (tobacco, alcohol and drugs) and psychological disorders (anxiety or depression) upon reaching adolescence. The lack of a significant improvement in this component at this point was expected because these concepts are difficult to grasp and it will therefore take time for this part of the intervention to yield results.

### Strengths and limitations

The main strength of this study is the use of a cluster-randomized, controlled trial design that allows for isolating the effects of the Program SI! intervention. This design was also preceded of qualitative studies on materials and strategies as well as pilot testing of the evaluation tools.

The intervention focuses on preschoolers and their immediate environment to ensure an effective intervention. Also, the intervention includes two cardiovascular-related components (understanding of the human body and heart, and management of emotions) that complement the traditional intervention areas of diet and physical activity. To achieve the behavioral modification, a multidisciplinary team designed intervention materials and strategies based on the trans-theoretical model of change but regrouping its predefined phases into our own definitions (knowledge, attitudes and habits).

Assessing participants’ adherence to the intervention is a great challenge of any community-based intervention study. The Program SI! is monitored by two supervisors (from the SHE Foundation and a school supervisor) who interact to ensure compliance and report on a yearly basis to measure school’s adherence to the intervention. A special effort is made to improve and maintain this active monitoring because of the intensity and the duration of the intervention.

An important limitation of this report concerns the lack of effect on parents and teachers. Parents received tips for healthy activities for the weekend that may have been insufficient to affect their own behavior. Likewise, teachers are initially trained in the Program SI! activities but it seems like it is difficult to retain their attention during the whole academic year. Achieving behavior change in adults has proven to be difficult in previous interventions, but we expect a larger effect by the end of the follow up time, at 3 years. A process evaluation to assess the appreciation of the feasibility and the subjective effectiveness of the program by parents and teachers may be needed. Finally, schools were selected from a group of schools that showed an interest on the Program SI! and therefore generalizability can be limited.

## Conclusion

The results after 1-school year intervention demonstrate that the Program SI! is an effective and feasible strategy for improving knowledge, attitudes and lifestyle habits among very young children. We expect these positive results to develop further by the end of the 3-year evaluation period and that they will validate Program SI! as a model for the introduction of healthy habits in early childhood, in line with public health priorities for healthy lifestyle promotion.

## Abbreviations

CVD: Cardiovascular disease; KAH-D: Diet knowledge, attitudes, and habits; KAH-PA: Physical activity knowledge, attitudes, and habits; KA-BH: Body and heart knowledge and attitudes; TEC: Test of emotional comprehension.

## Competing interests

The authors declare that they have no competing interests.

## Authors’ contributions

JLP conceptualized and designed the study, interpreted data, and drafted the manuscript. MSP carried out data analyses, interpreted data, reviewed and revised the manuscript. GSB coordinated and supervised data collection, and reviewed the manuscript. SP supervised the statistical analysis plan, interpreted data, and critically reviewed the manuscript. JR interpreted data, and critically reviewed the manuscript. VF conceptualized and designed the intervention program, and critically reviewed the manuscript. All authors approved the final manuscript as submitted.

## Pre-publication history

The pre-publication history for this paper can be accessed here:

http://www.biomedcentral.com/1471-2458/13/1208/prepub
